# Seven Years; It's Time for a Change

**DOI:** 10.1371/journal.pcbi.1002728

**Published:** 2012-10-04

**Authors:** Philip E. Bourne

**Affiliations:** 1 Department of Pharmacology, University of California San Diego, La Jolla, California, United States of America; 2 Skaggs School of Pharmacy and Pharmaceutical Sciences, University of California San Diego, La Jolla, California, United States of America

After seven years as the Editor-in-Chief of *PLOS Computational Biology*, I have decided to step to the side. It's time to bring in new leadership and a new vision. As scientists we generally do not learn a lot of management skills (a mistake in my opinion), but if I have learnt two management skills it is the value of enablement and to start planning for your successor on day one. Well, I did not start on day one, but Ruth Nussinov has been the Deputy Editor-in-Chief since October 2008, and she is an outstanding scientist and editor ideally suited to take over as editor-in-chief. So please welcome Ruth to this leadership role. The journal is in very safe hands. The journal staff, editors, and Ruth have not seen the last of me, however—this is just too much fun. As Founding Editor-in-Chief, I will continue to be involved with the journal, with a focus on special projects, helping Ruth and the team where I can, and, of course, continue as an author of both research articles and front matter, such as the Ten Simple Rules series.

In changing roles, I would like to make a few personal comments about the evolution of the journal and where it might go next. When Steven Brenner, Michael Eisen, and I founded the journal, we had a vision for how it would fill a gap between journals supporting purely computational methods and the array of experimental journals with the odd, token computational paper [1]. Thanks to you, the readers and authors, that vision has been realized beyond what we imagined, and I am very proud of how the journal is a voice for our broad and important community and at the same time helps build that community. The appearance of the journal in June 2005 was timely since it both propelled our field of science at a time when it was being recognized as a critical part of the life sciences, and made a strong statement about the importance of open access.

I must confess that when we started planning for the journal in 2004, support for open access seemed like the right thing to do, but it was not a major driver for me. That changed when, early on in my tenure, I realized open access is critical to maximizing the rate of scientific discovery. Supporting open access, and the new forms of scholarly communication it fosters, enriched my career and brought me into contact with many amazing people I would not otherwise have met. Emphasizing that enrichment, of the 22 invited lectures I gave in 2011, 18 were evangelizing about the importance of open access and open science, and four were directly related to my science. In my opinion, we have yet to see open access reach its full potential, but we will [2], and our journal will be poised to play an ever-increasing role as an exemplar for what is possible. In short, PLOS and this journal will continue to foster change, which maximizes the accessibility and comprehension of science. I am proud to continue to be a part of that.

It only remains for me to thank and acknowledge a variety of people who have all been so critically important in the past seven years. First and foremost are the approximately 150 editors who have worked tirelessly to shape and ensure a high-quality product over the years. There is no journal without community-driven efforts, which offer limited reward beyond a job well done. I can't mention everyone, but I must call out Karl Friston, who between 2005 and 2010 worked tirelessly to make computational neuroscience such a rich part of the journal, and Steven Brenner, Simon Levin, and Sebastian Bonhoeffer, who, since the journal's inception, have always responded with good advice.

Thanks are also due to Mark Patterson, who, until late 2011, was Director of Publishing at PLOS. Mark was critical to the success of the journal and to PLOS as a whole. We first began talking about the journal in May 2004, and he listened to, refined, and contributed to a lot of crazy ideas that define what the journal is today. To Catherine Nancarrow, who managed the journal from 2005 to 2010. A nicer and more dedicated person will not be found. In the current era of 140 character snapshots, her emails conferred a quality, beauty, and caring that you just don't see anymore. To Evie Browne, who contributed so much as Publications Assistant and Publications Manager between 2006 and 2009. Her spreadsheet analyses of how the journal was doing were amazing. To Andy Collings, who in various roles from Publications Assistant to Editorial Manager from 2005 to 2012 just made all ideas work, however outside the box they were. To Fran Lewitter, who, as Education Editor since the beginning, has created an important community jewel. To Scott Markel, who has been a voice of reason and an interface to the International Society of Computational Biology (ISCB) over the years. Finally, to all the other staff who have contributed over the past seven years: Emily Stevenson, Johanna Dehlinger, Helen Budd, Sheran Basra, and Cecy Marden, and the amazing current staff of Laura Taylor, Clare Weaver, and Chris Hall, all so well led by Rosemary Dickin and Theo Bloom.

PLOS is a family of journals and a family of people; in short, a family organization whose goal is to disseminate science in the most open and useful way possible. It is a successful organization able to recruit the best and most dedicated staff, and to adjust to its growing success and the success of open access itself. The world of scientific publishing will never be the same again because of PLOS and the people who drive it. I am proud to continue to be a member of this amazing family. There is no other publisher like PLOS and no other journal like *PLOS Computational Biology*.
